# What are the roles of retinoids, other morphogens, and *Hox* genes in setting up the vertebrate body axis?

**DOI:** 10.1002/dvg.23296

**Published:** 2019-04-25

**Authors:** Antony J. Durston

**Affiliations:** ^1^ Institute of Biology University of Leiden Leiden Netherlands

**Keywords:** axial patterning, gastrulation, *hox* genes, morphogens, retinoids, *Xenopus*

## Abstract

This article is concerned with the roles of retinoids and other known anterior–posterior morphogens in setting up the embryonic vertebrate anterior–posterior axis. The discussion is restricted to the very earliest events in setting up the anterior–posterior axis (from blastula to tailbud stages in *Xenopus* embryos). In these earliest developmental stages, morphogen concentration gradients are not relevant for setting up this axis. It emerges that at these stages, the core patterning mechanism is timing: *BMP*‐anti *BMP* mediated time space translation that regulates *Hox* temporal and spatial collinearities and *Hox‐Hox* auto‐ and cross‐ regulation. The known anterior–posterior morphogens and signaling pathways––retinoids, *FGF's, Cdx, Wnts*, *Gdf11* and others––interact with this core mechanism at and after space–time defined “decision points,” leading to the separation of distinct axial domains. There are also other roles for signaling pathways. Besides the *Hox* regulated hindbrain/trunk part of the axis, there is a rostral part (including the anterior part of the head and the extreme anterior domain [EAD]) that appears to be regulated by additional mechanisms. Key aspects of anterior–posterior axial patterning, including: the nature of different phases in early patterning and in the whole process; the specificities of *Hox* action and of intercellular signaling; and the mechanisms of *Hox* temporal and spatial collinearities, are discussed in relation to the facts and hypotheses proposed above.

## 1 INTRODUCTION

This review represents the conclusion of and harvest from (necessitated by old age) 20 years of intensively and obsessively pursuing a line of reasoning/scientific insight into the nature and ontogenesis of the vertebrate anterior–posterior (A–P) axial pattern. This main effort was preceded by other necessary and substantial phases devoted to learning vertebrate embryology and figuring out the way in to approach investigating and understanding vertebrate axial patterning and to learning how to approach developmental problems by using the elegant *Dictyostelium* system. Recent developments (8 recent articles from 6 major groups that generalize our *Xenopus* findings to mouse, chicken, and zebrafish) validate this line of reasoning and give confidence that it is correct.

## 2 WHAT IS THE ROLE OF A–P MORPHOGENS IN THE EARLIEST STAGE OF A–P PATTERNING. DO THEY ACT VIA STATIC CONCENTRATION GRADIENTS?

Are static morphogen concentration gradients relevant for the earliest A–P patterning (i.e., the stage when an axial pattern is first made starting in gastrulation)? Many previous models propose that retinoids and other morphogen signaling pathways have a role in patterning the vertebrate anterior–posterior (A–P) axis. A frequently used model has been that different threshold values on a morphogen concentration gradient specify different A–P axial positions (Carron & Shi, 2015; Godsave et al., 1998; Kiecker & Niehrs, 2001; Lamb & Harland, 1995; Lewis, Slack, & Wolpert, 1977; Meinhardt, 2009; Wolpert, 1969). But is this the only or even the primary direct mechanism in the earliest phase of vertebrate A–P axial patterning? Could a single static morphogen gradient pattern the whole body axis? I note that different known A–P morphogens are associated with different axial domains at different A–P axial positions (e.g., Godsave et al., 1998; Kiecker & Niehrs, 2001; Pownall et al., 1996), and that not many *Hox* genes (the determinants of axial positions posterior to the midbrain/hindbrain boundary) out of many tested in different organisms for regulation by the three best known A–P morphogen pathways (retinoid, *Wnt, FGF‐Cdx*) have actually been shown to be direct morphogen targets during early A–P patterning. Some clearly are not, but are however regulated indirectly, sometimes via *Hox‐Hox* interactions or other regulatory interactions (Faiella et al., 1994; In der Rieden, Vilaspasa, & Durston, 2011; Koop et al., 2010; Schubert, Holland, Laudet, & Holland, 2006). Only *Hox* genes at “decision points” (junctions between axial domains where morphogens act) and a few of the others are probably direct early A–P morphogen targets in the first phase of A–P axial patterning. Below, I puzzle out what the early role of morphogens actually is. Is a morphogen gradient the only mechanism involved in the first phase of axial patterning? Evidence is presented below that an entirely different mechanism: a timing mechanism (Section 3) is involved in the earliest steps in making the A–P axis. However, there is evidence that morphogen gradients are also important. Notably, they play a part in later detailed A–P patterning of the hindbrain (Section 8). One can, of course, not rule out that they also have other roles such as later respecifying and checking the initial pattern or acting concurrently with time–space translation (TST) to help specify the initial pattern. It is of course also quite possible that the thoughts in this article are wrong and that the initial axial pattern is specified solely by a morphogen gradient.

## 3 THE EARLY VERTEBRATE A–P AXIS IS GENERATED BY A TIMING MECHANISM, NAMELY BY *BMP*‐ANTI *BMP* DEPENDENT TST

### 3.1 Mechanistic clues from the early literature

**Figure 1:**
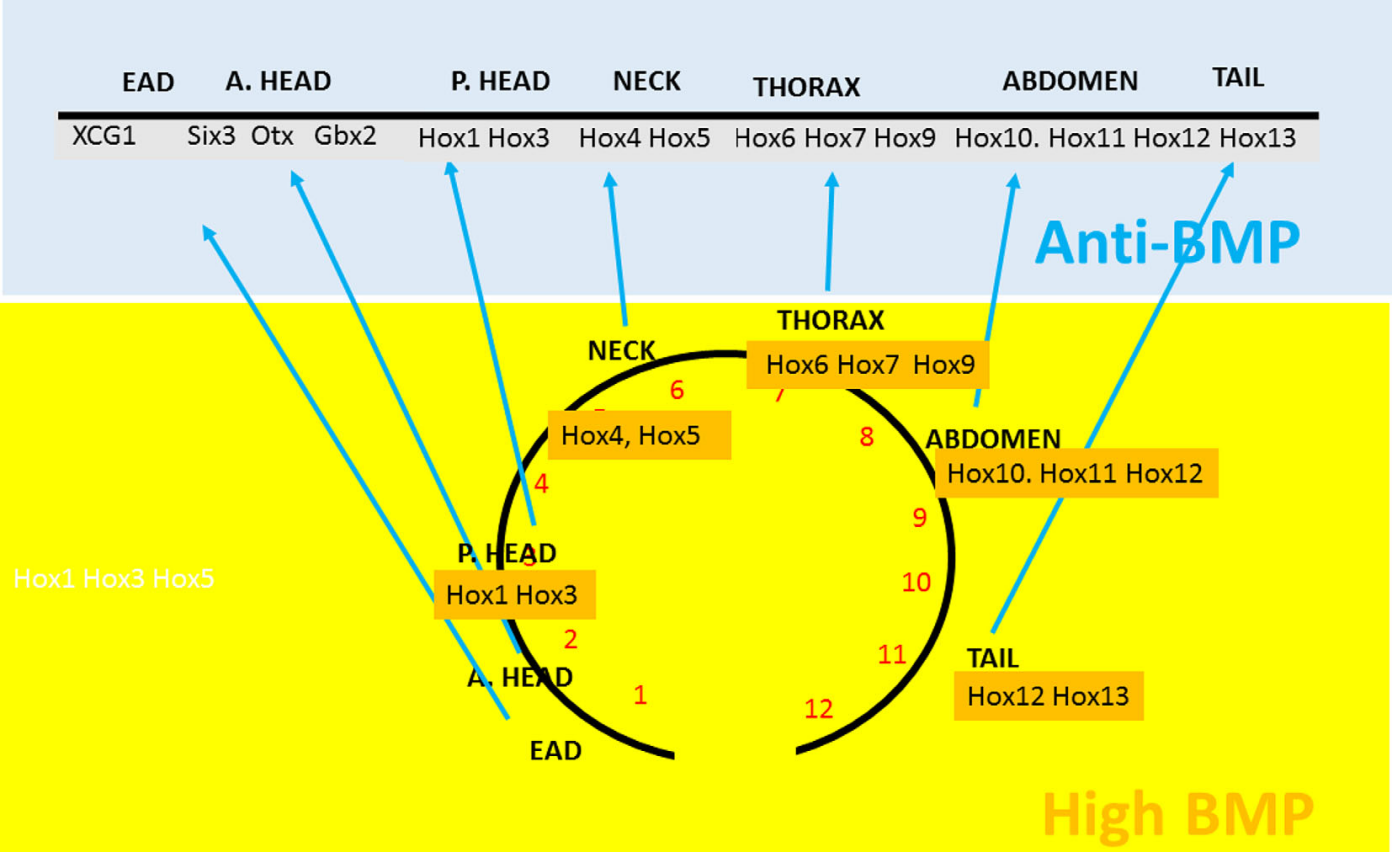
Timing, axial patterning, and time space translation. Above: The structure of the vertebrate A–P axis: domains with significant *Hox* genes and other markers. An unexpected element is introduced by the newly characterized extreme anterior domain (EAD), which makes the face. This is shown as the most anterior part of the straight axis. Actually, the anterior end of the dorsal A–P axis bends backward around to the ventral side of the embryo to face posteriorly like the handle of a walking stick (not shown). A. Head: anterior head (corresponding to telencephalon, diencephalon, mesencephalon). P. Head: posterior head (corresponding to anterior rhombencephalon, occipital somites). Neck: cervical somites, posterior rhombencephalon, Thorax: thoracic vertebrae, anterior spinal cord. Abdomen: Lumbar and sacral regions, spinal cord. Tail: coccygeal vertebrae, spinal cord. Above and below: Time space translation. A biological timer, represented by the clock face below, proceeds from 1 to 12 (red numbers). The timer starts with information needed for making the EAD, proceeds to the anterior head, then to posterior head, then to neck, then to thorax, then abdomen, then tail. The timer needs *BMP* to function, so occurs in tissues with high *BMP* (yellow/orange). Anti–*BMP* factors (blue) interact with the timer sequentially to freeze the identities of an A–P sequence of axial zones. In the axial sequence, the *Hox* genes are each both a component of the timer at their appropriate times and are sequentially involved in setting up the A–P sequence of axial zones. The genes involved in time space translation in the EAD‐head zones are unknown. The head and tail of the A–P timer are close together because of their representation on a clock face. No statement about molecular identities is intended

Nieuwkoop and collaborators first showed that the amphibian A–P axis is made in a timed manner. First the forebrain is induced, then progressively more posterior parts all the way back to the tail (Eyal‐Giladi, 1954; Nieuwkoop, 1952). Their studies and findings focussed on the A–P patterning of the central nervous system (CNS) and showed that the axial neural tissue is first specified as anterior (presumptive forebrain: telencephalon/diencephalon) and then sequentially posteriorised. This transformation involved first a conversion to presumptive mesencephalon, and subsequently to presumptive rhombencephalon, and then to presumptive spinal cord. These findings were confirmed by more recent studies in various vertebrates (Gamse & Sive, 2000, 2001; Stern, Charité, Deschamps, et al., 2006; Vasiliauskas & Stern, 2001; Wacker, Jansen et al., 2004). Recent work also shows that the head/brain is not the most anterior/early domain in the axis. There is actually a further rostral axial domain: the extreme anterior domain (EAD), newly discovered by Hazel Sive and colleagues, that lies anterior to the brain (Jacox, Sindelka, Chen, et al., 2014). The very recently characterized EAD, which becomes the ventral face and part of the pituitary gland (and the cement gland, in *Xenopus*), forms via ventral‐ward bending of the anterior end of the dorsal A–P axis so it faces backward ventrally like the handle of a walking stick (Jacox et al., 2014; Puelles, 2009; Figure 1).

### 3.2 The early A–P axis is made by TST

There is evidence that timed A–P axis formation is mediated by TST from gastrula stages onward (Durston & Zhu, 2015; Wacker, Jansen et al., 2004; Figure 1). There is a bone morphogenetic protein (*BMP*, a well‐known early morphogen) dependent timer (*Hox* temporal collinearity) in the nonorganizer (NO) mesoderm of the early embryo. Another embryonic region, Spemann's organizer (SO) emits anti‐*BMP* signals. Timed application of either an intact organizer or the organizer anti‐*BMP* signal *noggin* to the embryo at sequential stages blocked the timer at sequential A–P *Hox* values, and thereby fixed timed cell states sequentially. Either the treatment generated an anteriorly truncated axis with the truncation at sequentially more posterior positions for sequentially later treatments (implanted organizer: continuous anti‐*BMP* source) or it generated one or more A–P/*Hox* zones; sequentially later treatments gave sequentially more posterior zones (Wacker, Jansen, et al., 2004). We conclude that sequentially repeated interactions between the two embryonic parts lead to successive small populations of cells being fixed at sequential space/time points. Presumably, specific events including morphogenetic cell movements in the embryo or possibly *Hox* timed mesodermal cell ingression (Iimura & Pourquie, 2006), cause these sequentially timed/zoned populations to be arranged in and specify a patterned anterior early to posterior late spatial *Hox* sequence that becomes the initial A–P pattern. This TST mechanism was first demonstrated for the genesis of *Hox* pattern zones in the neck and more posterior parts of the body axis during gastrulation and later stages in *Xenopus* (Wacker, Jansen, et al., 2004). At these stages, the timer is initially in NO mesoderm in the gastrula and later in the NO mesoderm derivative presomitic mesoderm in the tailbud stage (Gont, Steinbeisser, Blumberg, & De Robertis, 1993). The fixed identity populations are in the dorsal paraxial mesoderm, which also contains and/or generates the timer population in presomitic mesoderm, and which is derived from NO mesoderm after convergence extension movements during gastrulation, as well as in dorsal neurectoderm, due to copying of identities from paraxial mesoderm to neurectoderm during neural transformation (Bardine et al., 2014; Mangold, 1933; Nieuwkoop, 1952). The timer in these stages manifests as *Hox* temporal collinearity. The A–P pattern of the fixed identity cell populations manifests as *Hox* spatial collinearity. The signals that fix time are anti‐*BMP* signals generated by the earlier organizer and later the chordaneural hinge. Cell populations acquire fixed identities as NO mesoderm moves dorsal‐ward to within range of the organizer due to convergence‐extension movements during gastrulation and later. This mechanism was first revealed in *Xenopus* but there is evidence that TST operates during gastrulation in other vertebrates from anterior early to posterior late timed stabilization. The conversion of a dynamically changing temporally collinear *Hox* sequence to a relatively stable spatially collinear axial *Hox* pattern of A–P positional information by anti‐*BMP* signals also operates in early chicken and zebrafish embryos (Dias, de Almeida, Belmonte, et al., 2014; Hashiguchi & Mullins, 2013). It has been shown that temporal collinearity in the chicken gastrula determines the order in which primitive streak cells migrate to the node (Denans, Iimura, & Pourquié, 2015; Iimura & Pourquie, 2006), that a population of dynamically changing primitive streak cells interacts with the a stable organizer derived population to generate the early axial pattern in mouse embryos (Wymeersch et al., 2018), and that *Hox* temporal collinearity during chicken gastrulation generates positional information (e.g., forelimb position) in later development (Moreau et al., 2018). These parallel and complementary findings in other vertebrate embryos establish that this timing mechanism is conserved.

Interestingly, the discoveries above define a believable role for the Spemann organizer, which is well known to be important in A–P patterning. I note that organizer‐less (ventralised) embryos show temporal but not spatial *Hox* collinearity and that reimplantation of an organizer reintroduces and fixes the spatial pattern exactly as predicted above (Wacker, Jansen, et al., 2004).

### 3.3 *Hox* function is part of the mechanism of TST

**Figure 2:**
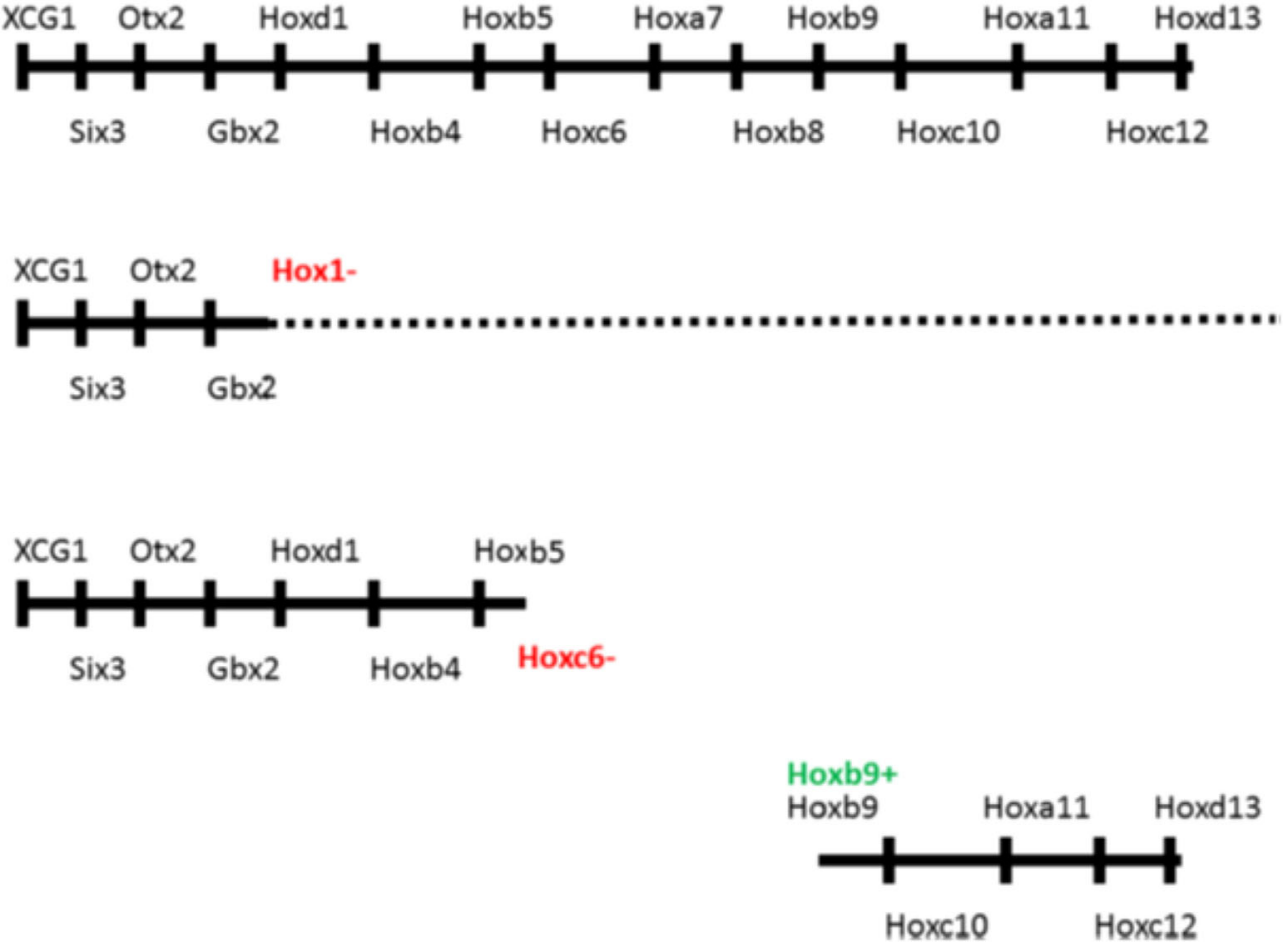
*Xenopus Hox* sequences for axial cascade phenotypes relating to domain boundaries. Above: Wild type *Hox* sequence. Next down: *Hox1* loss of function (LOF; all 3 *Hox1* genes knocked down by morpholinos (MOs). The axis from *Hox1* backward is compromised. The dotted line indicates there is still reduced residual expression for some posterior Hox genes. Next down: *Hoxc6* LOF (MO). The axis from *Hoxc6* backward is compromised/deleted. Next down: *Hoxb9* gain of function (GOF) by ectopic expression of *Hoxb9* in *Hox*‐free dorsalised embryos. A partial posterior axis is generated, starting with *Hoxb9*

During these stages, function of the *Hox* genes themselves is clearly a part of the timing and TST mechanism. The timing mechanism operates and the spatial *Hox* pattern is generated at least partly via autoregulation and cross‐regulation among the *Hox* genes, leading to a sequence of collinear interactions among them. This is thus a *Hox* cascade mechanism. The fact that *Hox* cascades are involved in specifying the axis is clearly demonstrated by the cascade of phenotypes generated by *Hox* gain and loss of function experiments in Xenopus and other systems (Faiella et al., 1994; Hooiveld et al., 1999; McNulty, Peres, Bardine, van den Akker, & Durston, 2005; Zhu, Spaink, & Durston, 2017a, 2017b; Figure 2). We have called this aspect of collinearity: macro‐collinearity (Durston, 2018). There are clearly three relevant noncell autonomous *Hox‐Hox* interactions.

#### 3.3.1 The induction of expression of more 5′ posterior *Hox* genes by more 3′ anterior *Hox* genes—referred to as posterior induction (PI)

Posterior induction (PI) is likely to be the basis of temporal collinearity (Faiella et al., 1994; Hooiveld et al., 1999; McNulty et al., 2005; Zhu et al., 2017a, 2017b). This regulatory mechanism works sequentially. The most 3′ anterior *Hox* genes are expressed earliest and they in turn are proposed to actively promote the expression of the next more 5′ posterior genes, and so on sequentially. In at least one case (induction of *Hoxb5* by *Hoxb4*; Hooiveld et al., 1999), this cascade reflects the fact that the 5′ nearest neighbor, *Hoxb5*, is a direct target of *Hoxb4* whereas more posterior *Hox* genes (i.e., *Hoxb9*) are not. PI is evident from the first expression of *Hox* genes in NO mesoderm during mid‐gastrulation (other *Hox‐Hox* interactions start later; Zhu et al., 2017a). It results in the development of a nested pattern of *Hox* genes. This interaction is therefore probably *BMP* dependent. It coincides with and could plausibly mediate *BMP* dependent temporally collinear *Hox* expression in *BMP*‐rich NO mesoderm in the gastrula, which is blocked at particular A–P *Hox* values by applying timed organizer signals or by timed applications of the *BMP* inhibitor, noggin, at particular stages during gastrulation (Jansen, Wacker, Bardine, & Durston, 2007; Wacker, Jansen, McNulty, Houtzager, & Durston, 2004; Wacker, McNulty, et al., 2004).

#### 3.3.2 Noncell autonomous autoregulation (Au)

*The copying of Hox gene* expression from cell to cell, clearly underlies vertical signaling during neural transformation, during which A–P information from paraxial mesoderm is copied to neurectoderm to fix its A–P patterning during gastrulation and later stages (Bardine et al., 2014). This form of autoregulation is dependent on *BMP* inhibition. It occurs in a tissue, the neurectoderm, that develops following the action of *BMP*‐inhibiting organizer signals including noggin. Autoregulation presumably also has other forms that are not all blocked by *BMP*.

#### 3.3.3 Posterior dominance (PD)

The suppression of expression or function of anterior *Hox* genes by more posterior ones, does not start until the end of gastrulation (Zhu et al., 2017b). This interaction includes, but is more than, the previous concept of “posterior prevalence” (PP; Duboule, 1991). The PP concept stipulates that interactions between *Hox* genes are at a functional level rather than at the level of mRNA regulation. PD is presumably involved in stabilizing mRNA *Hox* expression and making the spatial pattern (by TST), which starts from this stage. It is blocked by *BMP*.

The evidence that these three types of *Hox‐Hox* interactions mediate *Hox* collinearities and TST comes principally from gain and loss of function experiments, especially from the cascade phenotypes (Figure 2) that are sometimes seen (Faiella et al., 1994; Hooiveld et al., 1999; McNulty et al., 2005; Zhu et al., 2017a, 2017b).

Embryonic enhancers and *Hox* response elements that could mediate PI and Au (or similar interactions) have been reported previously (Ferretti et al., 2000; Kwan, Tsang, Krumlauf, & Sham, 2001; Maconochie et al., 1997; Manzanares et al., 2001; Popperl et al., 1995; Tümpel et al., 2007). PD is wider than the earlier concept of PP (Duboule, 1991). Unlike PP, PD involves regulation at the mRNA level.

### 3.4 How are the head and EAD patterned?

In the rostral head‐EAD part of the axis, an equivalent mechanism to that described above operates at still earlier stages. An anterior to posterior sequence of dorsal positional identities are fixed sequentially by anti‐*BMP* signals during zebrafish and *Xenopus* blastula‐gastrula stages (Hashiguchi & Mullins, 2013; Tucker, Mintzer, & Mullins, 2008; Zhu, Spaink, & Durston, 2017c). Presumably these identities derive from *BMP* inhibited early sequential fixation of identities determined by the *BMP* dependent timer. The identities are recognized using A–P markers that in some cases are expressed early and are therefore potentially part of the TST mechanism. In other cases, the markers are expressed late and are presumably therefore downstream of TST (Zhu et al., 2017c). The anterior‐early series of presumptive regions starts with the very early induced presumptive EAD, marked by expression of the cement gland gene, *XAG1* (late marker) and proceeds next with the presumptive telencephalon/anterior diencephalon, marked by expression of *Six3* (late marker), then goes on next to the presumptive diencephalon/mesencephalon, marked by *Otx2* (early marker) and to the presumptive anterior rhombencephalon (rhombomere 1; r1), marked by expression of *Gbx2* (earliest induced, most anterior retinoid induced marker, late marker), and next to presumptive mid‐rhombencephalon, marked by *Hoxd1* (early marker). These anterior zones are sequentially induced very early in development by timed anti‐*BMP* signals during mid‐blastula to mid‐gastrula stages, before and as the A–P *Hox* gene sequence starts to be expressed (Zhu et al., 2017c). This early sequence is thus contiguous with the later, more posterior *Hox* collinear expression. The mechanisms whereby TST takes place in this part of the axis are still obscure and there is little information about how the anterior part of the axis is specified. One possibility we consider is that the anterior states interact among themselves and with the *Hox* states in a very similar way as the *Hox* state zones interact among themselves (Durston, 2015; Zhu et al., 2017c). The anterior part of the axis develops exactly like the posterior *Hox* expressing part, by sequential insertion of zones expressing more and more posterior determinants (Gamse & Sive, 2001; Gamse and Sive, 2001). This is consistent with PI. Interactions including PD occur in this part of the axis, and between it and the *Hox* expressing domain (McNulty et al., 2005). There is thus possibly a continuous sequence of interactions between A–P determinants over the whole A–P axis.

### 3.5 What are the roles of *BMP* and anti‐*BMP*?

What are the roles of *BMP* and anti‐*BMP* in TST? We will deal with this in more detail below (Section 4), but briefly, *BMP* enables temporal collinearity. This is presumably because it enables a specific *Hox–Hox* interaction, namely PI, which is the basis of temporal collinearity. This is most likely the case because *BMP* is a signaling pathway that mediates or co‐regulates this inductive *Hox–Hox* interaction. Anti‐*BMP* blocks this signaling and therefore breaks the chain. It is known that downstream mediators of signaling via *DPP* (the *Drosophila* orthologue of *BMP*) are collaborators (noncooperatively binding coactivators) for a *Drosophila Hox* gene: *Ubx*. (Walsh & Carroll, 2007). The thoughts above are the background upon which I attempt to explain the role of morphogens in A–P patterning.

## 4 HOW DO A–P MORPHOGENS PARTICIPATE IN MAKING THE EARLY A–P PATTERN? INTERACTIONS BETWEEN THE EARLY TIMING MECHANISM AND A–P MORPHOGENS REVEAL A HIGHER LEVEL OF CONTROL, THAT GENERATES DISTINCT AXIAL DOMAINS

### 4.1 What is the relation of TST to axial domains? (and to A–P morphogens)

**Figure 3:**
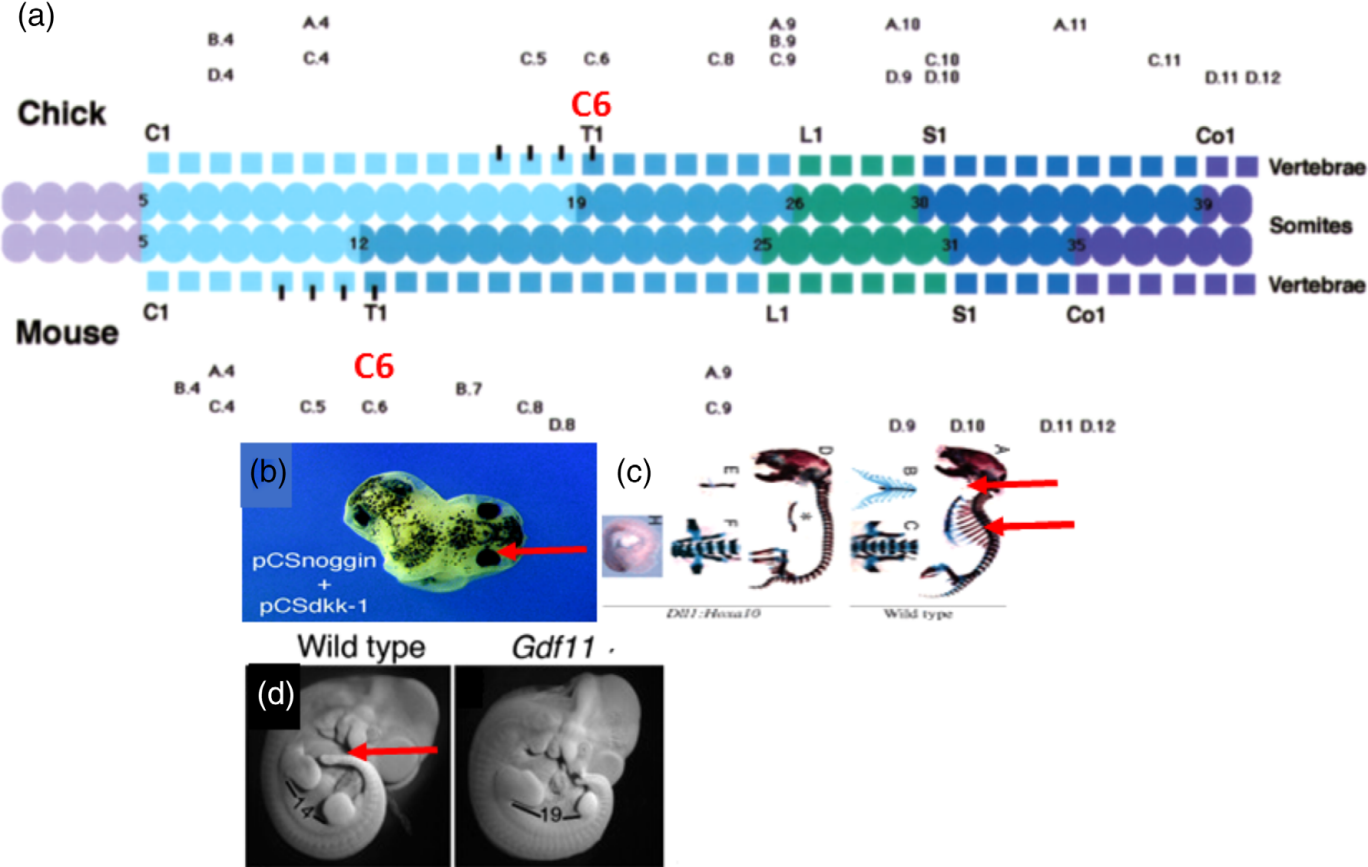
*Hox* genes, morphogens and boundaries between axial domains. (a) The neck‐thorax boundary and *Hoxc6* expression. Chick has a long neck (light blue) and short thorax (mid blue; 14 and 7 somites, respectively). Mouse has a short neck (light blue) and long thorax (mid blue; 7 and 14 somites, respectively). In each case, the *Hoxc6* anterior expression boundary (C6, marked in red) is at the neck/thorax boundary. Other vertebrates with different axial formulae (goose, *Xenopus*, zebrafish) show the same relationship. *Hoxc6* seems to be a special gene (Burke, Nelson, Bruce, Morgan, & Tabin, 1995). (b) Ectopic expression of the *Wnt* inhibitor *Dickkopf (Dkk‐1)* in the presence of the anti‐*BMP* dorsaliser, noggin, causes *Xenopus* embryos to develop head structures only (eye red arrowed); the anterior head‐posterior head boundary is blocked (Glinka et al., 1998). (c) Ectopic expression of *Hoxa10* in the mouse. Right: Normal mouse skeleton showing thoracic ribs (marked by red arrows). Left: *Hoxa10* GOF skeleton, showing no ribs. The whole thorax has become abdominal (lumbar vertebrae; thorax‐abdomen boundary deleted; Carapuço, Nóvoa, Bobola, & Mallo, 2005). (d) Normal and *Gdx8−/−* mice. The normal mouse (left) has a tail (red arrow). The *Gdx−/−* mouse essentially does not. The abdomen‐tail boundary is blocked (Jurberg, Aires, Varela‐Lasheras, Novoa, & Mallo, 2013)

**Figure 4:**
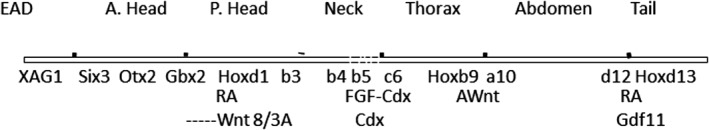
Growth factors and the axial domains. The anterior head (A. Head)/posterior head (P. Head) boundary is influenced by active retinoids/retinoic acid (RA) and *Wnt* (8 or 3A). Both turn posterior head on. The neck/thorax boundary is influenced by RA (thorax off) and *FGF/Cdx* (thorax on). The thorax/abdomen boundary is influenced by *Wnt*. The abdomen/tail boundary is influenced by *GDF11* (tail on) and RA, which blocks the transition of abdomen to tail, that is, it changes tail to limbs or truncates the axis

What is described in Section 2 is the very earliest, basic, core mechanism for making the vertebrate A–P axis. Its *BMP*/anti‐*BMP* dependent nature presumably accounts for at least part of the well‐known connection between A–P and dorsoventral (D‐V) patterning (since *BMP* is best known as a ventralising morphogen and anti‐*BMP* factors are best known as dorsalising morphogens (Bier & de Robertis, 2015; Lane & Sheets, 2002). What, however, are the connections between this mechanism and the known A–P morphogens? These are presumably implicated in later events in A–P patterning, such as hindbrain specification. There is, however, also a higher level of control in the earliest patterning phase that involves A–P morphogens as well as TST. The vertebrate body axis is obviously divided into domains, such as the head, neck, and thorax. (Figures 1, 3, 4). A novel element is introduced in vertebrates by the recently characterized most anterior EAD that makes the vertebrate face (Section 3 above). This subdivision into domains enables development of a functional animal. The axial domains are made up of different tissue types. The A–P axis is defined most obviously in the dorsal tissues of the early embryo, notably, in dorsal paraxial somitogenic mesoderm and in the neuroectoderm (presumptive CNS). Each of the more posterior primary axial domains is defined in the paraxial mesoderm, leading to development of domain‐specific types of vertebrae, namely, occipital (posterior head), cervical (neck), thoracic, abdominal (lumbar and sacral), and tail (coccygeal). The developing CNS is less obviously divided. There are several developing regions in the brain (telencephalon, diencephalon, mesencephalon, rhombencephalon). The most posterior of these (rhombencephalon = hindbrain: r2–r8) corresponds molecularly (via *Hox* expression) to the occipital and cervical regions of paraxial mesoderm. The post‐hindbrain CNS (developing spinal cord) has a collinear sequence of *Hox* anterior expression boundaries similar to the paraxial mesoderm, but is not obviously divided into domains. Some domains are also subdivided into what are presumably tissue specific subdomains, for example, the division of presumptive abdominal paraxial mesoderm into presumptive lumbar and sacral regions. Mesodermal domains can also be segmented metamerically from the cephalic region back to the tail into somites and neural domains in the rhombencephalon and perhaps more anterior regions into neuroectodermal rhombomeres and prosomeres (Puelles, 2009). For the analysis below, I propose the following domains: (1) EAD; (2) anterior head, corresponding to telencephalon, diencephalon, mesencephalon; (3) posterior head, corresponding to anterior rhombencephalon and occipital somites; (4) neck, corresponding to posterior rhombencephalon and cervical somites; (5) thorax; (6) abdomen, corresponding to lumbar and sacral somites; and (7) tail. These axial domains are distinguished by axial morphogens, as described in Section 4.3 below.

Like the A–P determinants that specify positions along the axis, the axial domains are specified sequentially in time: first the EAD and head, then the neck, then the thorax, then abdomen and tail. This timing appears to require interactions between successive local A–P determinants and A–P signaling pathways. For example, turning on the *Gbx2* and *Hox* genes in the presumptive rhombencephalon apparently requires the presence of the immediately neighboring anterior axial zone, that is, the diencephalon/mesencephalon or signals or determinants derived from them. In particular, *Otx2* expression has been implicated. This sequential process was first discovered by Nieuwkoop and collaborators for early neuroectoderm, which is first specified as anterior brain (activation) and then in sequentially later steps is progressively posteriorised (transformation; Eyal‐Giladi, 1954; Nieuwkoop, 1952). Nieuwkoop and colleagues discovered that anterior embryonic neurectoderm can be posteriorised to different posterior states, but it is precursor tissue, the early non‐neural embryonic ectoderm, cannot. It appears that specific neurectodermal determinants are required.

### 4.2 What are the relations of axial domains to *Hox* genes?

Different axial domains and the transitions between them are associated in the early embryo with expression of particular axial determinants. In the trunk‐tail part of the axis, these determinants are the *Hox* genes (Figure 3a). The anterior boundaries of earliest *Hox1* gene expression are always just after the midbrain‐hindbrain transition, and correspond to the anterior boundary for occipital PM (Burke et al., 1995; Gaunt, 2000; Hashiguchi & Mullins, 2013; McNulty et al., 2005). The anterior boundary for *Hoxb4* is close to the boundary between posterior head and neck and the occipital/cervical transition in the PM. The anterior boundary for the earliest *Hoxc6* expression is always at the cervical‐thoracic transition in PM (Burke et al., 1995; Gaunt, 2000; McIntyre et al., 2007; Zhu et al., 2017a, 2017b). The anterior boundary for *Hoxa10* is almost always at the thoracic‐lumbar transition (Burke et al., 1995; Carapuço et al., 2005; McIntyre et al., 2007; Woltering et al., 2009). These relationships are evolutionarily conserved. The anterior boundary of *Hoxc6* expression coincides with the anterior boundary of the thorax in five different vertebrates examined (zebrafish, *Xenopus*, chicken, goose, mouse; (Burke et al., 1995). These vertebrates have different axial formulae. In mouse, the cervical‐thoracic transition occurs after 7 cervical vertebrae (derived from successive somites) and the thorax is 14 vertebrae long. In chicken, this transition occurs after 14 cervical vertebrae and the thorax is 7 vertebrae long. These observations led to the suggestion that *Hoxc6* regulates the neck/thorax transition. Support for this idea comes from evidence that *Hox* genes expressed at domain boundaries do indeed play a role in switching between domains. *Xenopus Hoxc6* loss of function cuts off the axis and the A–P *Hox* sequence at the anterior thoracic boundary, which is the *Hoxc6* anterior expression boundary (Zhu et al., 2017b). Mouse *Hoxa10* gain of function converts mouse thorax to lumbar, which starts at the *Hoxa10* anterior boundary (Figure 2; Carapuco et al., 2005). *Xenopus Hox1* loss of function compromises the axis and blocks or diminishes the whole *Hox* expression sequence from the anterior head‐posterior head boundary backward (McNulty et al., 2005), as would be expected according to this model. The fact that the *Hoxc6* mouse knockout phenotype is milder than the *Xenopus Hoxc6* morpholino (MO) knockdown phenotype (cf. McIntyre et al., 2007; Zhu et al., 2017b) is not unexpected considering that murine knockouts, but not (*Xenopus*) MO translation knockdowns, are influenced by genetic compensation (Rossi et al., 2015). It should be noted that in some vertebrates, the usual evolutionarily conserved *Hox* expression boundaries are not retained. These organisms have unusual, aberrant body plans. For example, snakes and caecilians have an elongated thorax but no longer have coincident abdomen/thorax boundaries and *Hoxa10* anterior expression boundaries (Woltering et al., 2009).

### 4.3 What is the relation of axial domains to A–P signaling pathways? The notion of decision points

#### 4.3.1 Switches in the action of signaling pathways occur at boundaries between axial domains

The time–space localized positions, where one axial domain switches to the next, have been called “decision points” (Durston, 2015). These decision points are associated with and in some cases have been shown to require, switches in the activities of A–P morphogens and their signaling pathways during early development. Switching from anterior brain/head to more posterior rhombencephalon/posterior head requires the onset of retinoid signaling from mesoderm that acts on the neuroectoderm (Godsave et al., 1998; Schubert et al., 2006). It also requires wingless‐int (*Wn*t, a well‐known early embryonic growth factor) signaling. *Wnt* signals are mesodermal in origin, arising in *Xenopus* from the NO mesoderm or from the mouse equivalent, the primitive streak (Elkouby et al., 2010; In der Rieden et al., 2011; Kiecker & Niehrs, 2001; Neijts et al., 2016; Niehrs, 1999). Switching from neck neuroectoderm (presumptive rhombencephalon) to thorax neuroectoderm (presumptive anterior spinal cord) requires a switch from retinoid signaling (on neck/off thorax) to fibroblast growth factor (*FGF*, a well‐known early embryonic growth factor) together with *Cdx* signaling (off neck/on thorax; Bel Vialar et al., 2002; Godsave et al., 1998; Pownall et al., 1996). These specific cases are described below. Switching from abdomen to tail requires the onset of *Gdf11* signaling, an embryonic *TGFb* family factor (Jurberg et al., 2013; Figures 3 and 4). On the other hand, late stage retinoid gain of function also affects this transition. It can cause genesis of a cluster of limbs instead of a tail at this boundary in some amphibians (Mahapatra & Mohanty‐Hejmadi, 1994). Retinoids can also truncate the posterior axis in the mouse (Kessel & Gruss, 1991). In addition, there are two other important signaling pathway/domain associations. Development of the EAD requires *kinin–kalikrein* signaling. This may, however, be important only for later events rather than the earliest decisions (Jacox et al., 2014). Development of the head and EAD also requires inhibition of *Wnt* signaling, which is required for more posterior development from the neck backward, as well as of *BMP* signaling (Glinka et al., 1998; Kiecker & Niehrs, 2001). It should be noted that certain axial boundaries like the midbrain/hindbrain boundary (Rhinn & Brand, 2001) have long been considered to have organizer functions.

#### 4.3.2 The mode of action of these signaling pathways can involve activating *Hox* genes

At least some of the more posterior signaling pathways each activate particular *Hox* genes at and after “decision points.” Retinoids in *Xenopus* and mouse activate *Hox* paralogue groups 1–5 (neck) but not necessarily more posterior *Hox* genes (Bel Vialar et al., 2002; Godsave et al., 1998). In *Amphioxus*, retinoids activate *Hox1‐6* (Schubert et al., 2006). *FGF‐Cdx* activates at least *Hox6‐9* in *Xenopus* and mouse (thoracic) but not *Hox1‐5* (neck; Bel Vialar et al., 2002; Pownall et al., 1996). *Wnt8* activates at least *Hox* paralogue groups 1‐6 in *Xenopus* (In der Rieden et al., 2011).

The axial patterning process works via TST. It starts with temporal collinearity in NO mesoderm and goes on to spatial collinearity in neurectoderm and axial mesoderm. It is important to know at what stage of this process signaling pathways impact decision points and TST. For retinoids, these are known to exert an effect on axial neuroectodermal expression but not much is known about earlier effects (Godsave et al., 1998; Schubert et al., 2006). Wnt8 is known to impact early, temporally collinear *Hox* expression in *Xenopus* NO mesoderm or in mouse primitive streak (In der Rieden et al., 2011; Neijts et al., 2016). *FGF‐Cdx* is known to impact neuroectodermal spatial collinearity, as with retinoids (Bel Vialar et al., 2002; Epstein, Pillemer, Yelin, Yisraeli, & Fainsod, 1997; Pownall et al., 1996), but *Cdx* also impacts early temporal collinearity in the early mouse primitive streak (Neijts, Amin, van Rooijen, & Deschamps, 2017).

### 4.4 Signaling pathway–domain interactions are mediated at least partly by *Hox–Hox* interactions

*Hox* collinearities require *Hox–Hox* interactions. In asking how a signaling pathway can interact with collinearity, it is useful to bear this fact in mind. There is information about the effects of three signaling pathways on *Hox* collinearity.

#### 4.4.1 The effects of retinoids on collinearity: the anterior head‐posterior head junction

It is known in human embryonic stem (ES) cells (an embryo‐like system) that retinoids induce temporally collinear (TC) expression of *Hox* genes from *Hox1* to *Hox10* and that the TC sequence can be broken by antisense RNA knockdown for two of the 3′ early anterior *Hox* genes that were tested: *Hoxb1* and *Hoxb3*. Both knockdowns cut off the posterior part of the TC sequence in all four *Hox* clusters from the paralogue position of the gene studied (Faiella et al., 1994). This regulatory interaction has also been investigated in embryos of the cephalochordate *Amphioxus*, which is closely related to vertebrates. Although it has only one *Hox* cluster, it is believed to share similar regulatory mechanisms. Treatment of early *Amphioxus* embryos with the retinoid antagonist BMS009 (retinoid loss of function) gives a phenotype that is closely similar to that for MO‐induced loss of function for *Amphi‐Hox1*. It posteriorises expression of *Amphi‐Hox1‐6*. On the other hand, retinoid gain of function anteriorises expression of *Amphi‐Hox1‐6* (Schubert et al., 2006). The similarities between the retinoid loss of function and *Amphi‐Hox1* loss of function phenotypes led to the suggestion that retinoid action works in this system via induction of *Amphi‐Hox1,* as in the human ES cells (Faiella et al., 1994). However, only *Amphi‐Hox1* and *Amphi‐Hox3* turned out to be direct retinoid targets in early *Amphioxus* development (Koop et al., 2010). The other *Hox* genes were induced indirectly through cross‐regulatory interactions, probably initiated by *Amphi‐Hox1* and *Amphi‐Hox3*. Comparable results were obtained in *Xenopus*, in which *Hox1* paralogue group loss of function compromises expression of all more posterior *Hox* genes examined (McNulty et al., 2005), similar to results obtained in human ES cells and *Amphioxus* (Faiella et al., 1994; Schubert et al., 2006). We conclude that retinoids induce *Hox* genes via *Hox1* and *Hox3* in *Amphioxus*. There is information about which mammalian and human *Hox* genes have retinoid response elements (Langston, Thompson, & Gudas, 1997; Mainguy et al., 2006). The earliest, most anterior RA responsive gene is likely to be the decision point gene for the vertebrate head–neck retinoid switch. Probably this is *Gbx2* or its local upstream regulator. I note further that all human and mouse hindbrain *Hox* genes have been shown by bioinformatics to contain putative retinoid response elements (RAREs), but it is not known when, where or even if these are active (Mainguy et al., 2003). Mouse *Hox1* genes are also known to be regulated early on by functional RAREs (Huang, Chen, & Gudas, 2002; Langston et al., 1997). It seems certain that retinoids directly regulate *Hox1* genes early on. They also show direct early neural regulation of *Hoxb4* and *Hoxd4,* the two *Hox4* genes that are expressed closest to the posterior head/neck boundary (Gould, Itasaki, & Krumlauf, 1998; Morrisona et al., 1995; Nolte, Amoresc, Kova'csb, Postlethwaitc, & Featherstone, 2003; Zhang, Kovács, & Featherstone, 2000). Detailed analysis of the mouse *Hoxb4* and *Hoxd4* genes showed that they are evidently regulated by a combination of a retinoid input and a *Hox* input (Serpente et al., 2004). The *Hox* input also feeds back on retinoid signaling via *RARB* (Serpente et al., 2004). The posterior head/neck boundary is possibly thus also retinoid regulated. Also, whereas the reports above concern retinoid action in the developing neuroectoderm and endoderm (pharynx), there are various indications that retinoids also function in patterning the early mesoderm during somitogenesis, another TST process.

#### 4.4.2 The effect of *Wnt* on collinearity: the anterior head‐posterior head junction

*Wnt* action has been studied in *Xenopus*, where *Wnt8* loss of function and ectopic expression were used to examine *Wnt* regulation of *Hox* genes. *Wnt 8* induces *Hox* genes (*Hoxa1*, *b1*, *d1*, *b4*, *d4*, *c6* tested; In der Rieden et al., 2011). Only *Hox1* genes were directly induced (induced in the presence of cycloheximide). The others were induced indirectly, probably via *Hox1*. The sample of *Hox* genes examined here was small but it is possible that, unlike the situation with retinoids, *Wnt* only directly induces *Hox1* genes, not multiple genes in the posterior head and neck domains.

#### 4.4.3 The effect of *FGF‐Cdx* on collinearity: The neck‐thorax junction

This has been studied in *Xenopus*, chicken, and mouse. *FGF‐Cdx* induces neuroectodermal expression of all thoracic *Hox* genes but none of the neck or posterior head *Hox* genes studied in *Xenopus* and chicken. *Hoxb6*, *Hoxc6*, *Hoxa7*, *Hoxb7*, *Hoxb8*, *Hoxb9,* but not *Hoxb1*, *Hoxb3*, *Hoxb4* or *Hoxb5*, were induced by FGF (Bel Vialar et al., 2002; Pownall et al., 2006). *Cdx* also represses anterior *Hox* genes as well as inducing the posterior ones (Epstein et al., 1997). It is known that expression of all of the thoracic *Hox* genes tested is deleted by loss of the earliest, most anterior thoracic *Hox* gene induced, *Hoxc6*, in *Xenopus* (Zhu et al., 2017b). It is therefore possible that all these thoracic *Hox* genes are induced indirectly via *Hoxc6*, in *Xenopus* at least. These results are consistent with the concept that *Hoxc6* is an important direct *FGF‐Cdx* target and also support the idea presented above that it is a key decision point *Hox* gene for the transition from neck to thorax. We note however that *Hoxc8* is also directly induced by *Cdx* during early embryogenesis (Schyr, Shabtai, Shashikant, & Fainsod, 2012).

#### 4.4.4 General conclusion

**Figure 5:**
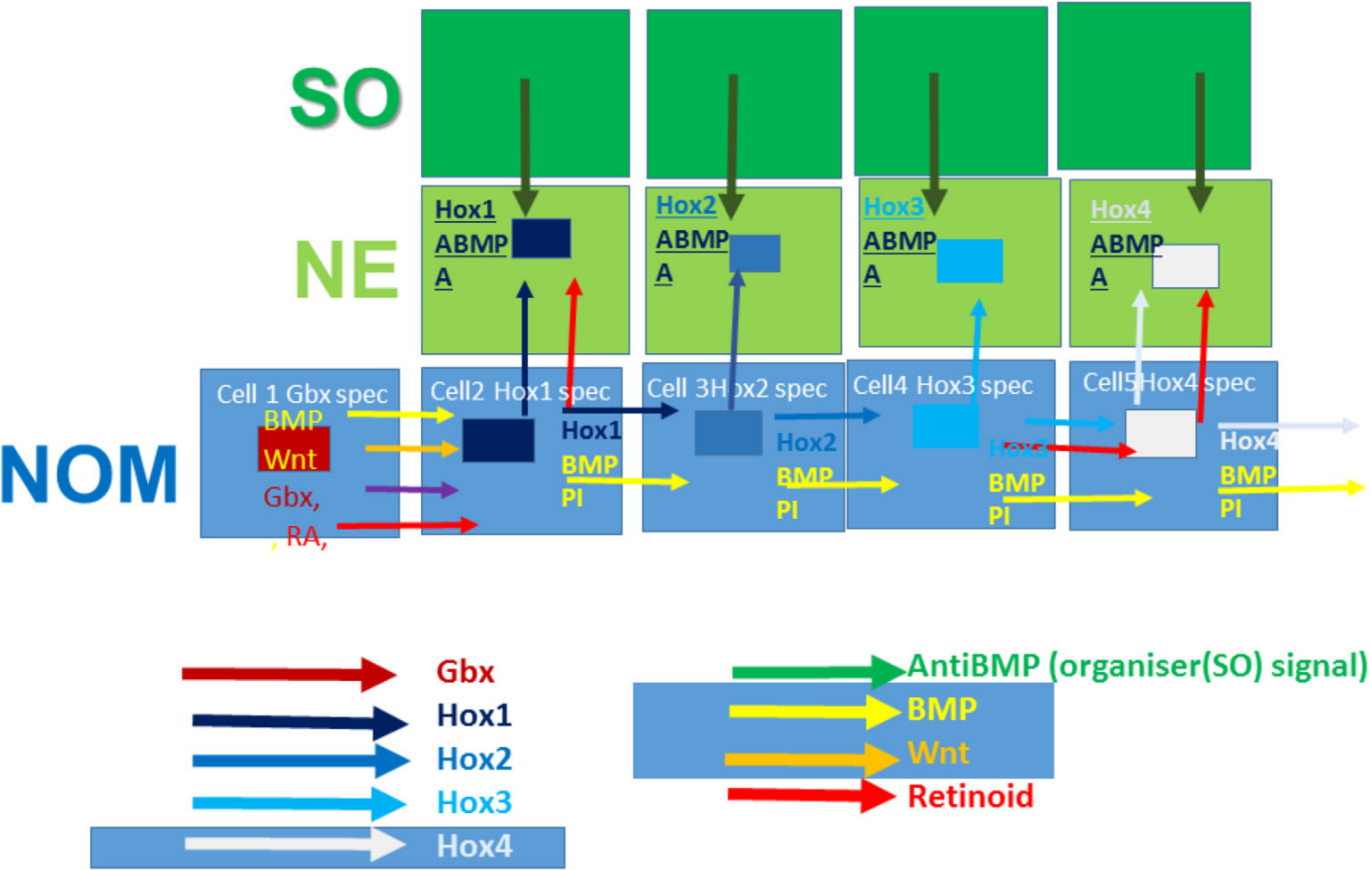
Intercellular signaling in *Hox* collinearity and TST *BMP* signaling (yellow arrows} is permissive for noncell autonomous *Hox‐Hox* PI signaling in NO mesodem (NO mesoderm cells are big blue oblongs). *Hox* expression occurs in each cell with different specificities. The shade of blue in the small rectangles (nucleus) indicates which specificity. (1) First blue cell to the left: *Gbx2* specificity (mauve). *Gbx* is not known to be expressed in NO mesoderm, only in neurectoderm (NE). The mauve color represents the underlying A–P level specificity. The mauve nucleus emits a specific signal (mauve arrow) to cell 2. (2) *Hox1* specificity, nucleus darkest blue shade. This emits a *Hox1‐*specificity signal (dark blue arrow) to cell 3 (which becomes *Hox2* specificity (mid blue nucleus). (3) Cell 3 emits a *Hox2‐*specific signal to cell 4 (mid blue arrow), etc. The different colored blue arrows indicate that cells emit specific *Hox* signals to neighboring cells. The specificity of the signal is the same as the expression specificity of the emitting cell. The response is to develop the next more posterior specificity (PI). One signaling mechanism that could accomplish this is that *Hox* homeoproteins could be conveyed nonspecifically from cell to cell by Prochiantz transfer (i.e., if they are expressed, they are transferred; Joliot & Prochiantz, 2004) and that the specificity of the response is determined intracellularly by *Hox* transcription factor specificity in the nucleus. All NO mesoderm‐NO mesoderm interactions also require a yellow arrow (*BMP*). This determines the specificity of the interaction as PI. The *Gbx2‐Hox1* transition also requires *Wnt* signaling (orange) and retinoid signaling (red). These enable the anterior head‐posterior head transition. The blue cells also signal vertically to light green cells (NE, neurectoderm). This represents specific *Hox* signaling (Au) from NO mesoderm to NE, copying the same specificity. In all cases, this also requires a dark green signal from neighboring dark green cells (Spemann Organizer: SO). These green *BMP* inhibitions specify this interaction as Au and not PI. Please note that the position of the dark green SO cells is not pictorially accurate. All that is indicated is that these cells are close to and thus in range of the NE. In the first case (copying *Hox1* specificity), the copying also requires retinoid signaling (red arrow) as well as the specific *Hox* signal. This represents retinoid signaling at the anterior head–posterior head decision point. Red arrows are also shown for '*Hox4* NO mesoderm‐NE and for *Hox3* NO mesoderm‐ *Hox4* NO mesoderm to indicate the retinoid requirement for vertical signaling is not exclusively confined to the anterior head‐posterior head decision point. I note that vertebrate *Hox*b4 and *Hoxd4* expression at the *Hox4* posterior head‐neck decision point is known to be depend directly on retinoid signalling. Mauve rectangle and arrow indicate the expression of and specific signaling from the last anterior head determinant (*Gbx*). Orange arrow: *Wnt* signaling: Required for NOM mesodermal transition through the anterior head–posterior head decision point. The proposal is that *Hox1* is turned on in the NO mesoderm by the last anterior head determinant and *BMP*, *Wnt* and retinoids, and is then signaled from NO mesoderm to NE via *Hox*, *BMP* inhibition and retinoids. The scheme below the figure is a color key for the signaling arrows. It should be noted that *Gbx1* level i I is presented in this figure as the most posterior A‐P level in the anterior head domain. This is for purposes of explanation and discussion only. In fact, *Gbx2* is more probably at the most anterior or a very anterior level in the posterior Head domain

The evidence above emphasizes that collinearity can be initiated by the retinoid, *Wnt* and *FGF‐Cdx* signaling networks but also involves *Hox‐Hox* interactions. The data indicate that different signaling pathways interact with the *Hox* collinearity sequence at and after pathway specific decision points. A decision point is presumably located at the first determinant/*Hox* gene at which a new signaling pathway first becomes relevant for PI or a different interaction. In fact, A–P signaling pathways may interact, presumably as collaborators or cofactors, with multiple *Hox* genes in a domain. The important point is that a new domain can acquire a new signaling pathway and may shut off a previous one. Clearly, some *Hox* genes also are induced indirectly by A–P signaling pathways rather than being direct targets (Figure 5).

## 5 EXCEPTIONAL SIGNALING PATHWAY/*HOX* INTERACTIONS

The evidence above speaks for the involvement of signaling pathways at and after “decision points”. There are at least three possible exceptional cases where signaling pathways may have a different role.

### 5.1 *BMP*‐anti *BMP*

Why does *BMP* permit temporal collinearity (TC) generally? And, why does anti‐*BMP* not permit it, but instead fosters spatial collinearity (SC) and vertical signaling autoregulation (Au)? TC is putatively mediated by PI, a noncell autonomous *Hox–Hox* interaction. There are in principle at least two ways in which signaling pathways could interact with PI and temporal collinearity specifically and with the *Hox‐*macro‐collinearity mechanism (Durston, 2018) in general. One is that they may be external to this mechanism or be limited to action on specific *Hox* genes, in which case they could interact with this mechanism and participate in triggering *Hox* expression at and after decision points, as above. The second is that they may act as coactivating signaling pathways, generally co‐mediating noncell autonomy of a particular collinear *Hox–Hox* interaction. It is possible that *BMP* co‐mediates PI. It differs from a decision point morphogen in that it is relevant for the whole axial *Hox* sequence as well as the sequence of head determinants. Then, how is it possible that high *BMP* levels support temporal collinearity, while *BMP* inhibits development of spatial collinearity and autoregulatory vertical signaling of NO mesoderm *Hox* identities from mesoderm to neuroectoderm? It is logical that *BMP* acts as a collaborator for noncell autonomy of the PI interaction, which occurs only in the presence of high *BMP* concentration and specifically mediates temporal collinearity. Removing *BMP* will then block this PI interaction and possibly convert it to something else, possibly autoregulation or posterior dominance (PD), thus breaking the TC cascade and stabilizing *Hox* expression (Figure 5). In fact, autoregulation in the context of vertical signaling and PD are both known to be inhibited by *BMP*. The *Drosophila BMP* orthologue, *DPP*, is known as a *Hox* upstream regulator for several different *Hox* genes in the developing midgut (Staehling‐Hampton & Hoffmann, 1994). *DPP* is also known to collaborate with *Ubx* (Walsh & Carroll, 2007). One could ask: is *BMP* the only factor mediating PI signaling? We suspect not because PI depends on complex specificity that could not easily be delivered by *BMP* alone (discussed below). TC is sequential by direct induction of expression going from one *Hox* paralogue group (PG) primarily to other *Hox* genes close to it, for example, to its immediate 5′ neighboring PG. It can be assumed, therefore, that PI does the same. In the one case that has been tested, it does: *Hoxb4* induces *Hoxb5* directly but *Hoxb7* and *Hoxb9* indirectly (Hooiveld et al., 1999). Therefore, we suspect each *Hox* gene induces only its 5′ neighbors. PI could perhaps also function for all of the head and EAD determinants described above, but there is little information. PI does function for all *Hox* genes tested, covering at least from *Hox1* to *Hox9. Hox10‐13* have not yet been tested but there is no reason to think they behave differently. There are, however, at least two other *Hox/*signaling pathway interactions that respect domains but do not obviously fit with the decision point concept.

### 5.2 Posterior *Hox* genes: *Hox9‐13*: Abdomen‐tail collinearly repress *Wnt* signaling

*Hox* paralogue groups *9–13* repress *Wnt* with collinearly increasing strength and thereby repress continuing growth of the axis that depends on the *Wnt* target *Brachyury* (Denans et al., 2015). This gives pause for thought. It is possible that in the abdomen and tail domains of the axis, noncell autonomous PI involves anti‐*Wnt* pathway signaling. Possibly this is synergistic with *BMP* signaling in mediating PI. Possibly it replaces *BMP*. Anti‐*Wnt* appears to be a decision point pathway that is required for PI by every posterior gene in this part of the axis. Cumulative build‐up of anti‐*Wnt* factors during sequential induction of more and more posterior *Hox* genes by PI would generate increasing anti‐*Wnt* action.

### 5.3 Somitogenesis: A second TST mechanism interacts with *Hox* patterning

During somitogenesis, mesodermal segments (somites) are generated by interaction of a temporal oscillation (somitogenesis clock) in presomitic mesoderm with an anterior to posterior traveling wave front that fixes oscillation phase and generates spatially periodic somites in a sequence from anterior to posterior (Palmeirim et al., 1997). The somitogenesis oscillation clock involves the *Notch* and *Wnt* pathways and is maintained by FGF. The fixation wave involves retinoid signaling. This is a different kind of TST mechanism from that described above and it is linked to A–P patterning. Possibly the fixation wave front could be viewed as a traveling decision point. *Hox* expression interacts with somitogenesis such that boundaries between particular somite numbers are also anterior expression boundaries for particular *Hox* genes in the mesoderm. This interaction can apparently be mediated by interactions between *Hox* genes and the *Notch* pathway. Downregulating the *Notch* pathway somitogenesis gene, *Delta2,* reduces mesodermal expression of *Xenopus Hox* genes as well as blocking somitogenesis (Peres, McNulty, & Durston, 2006). *Hoxd1, Hoxb4, Hoxc6*, and *Hoxb9* were downregulated from gastrulation onward. Similarly, *Notch* pathway *RBPjk* deficient mouse embryos also downregulate *Hox* genes in presomitic mesoderm, supporting the same conclusion (Zakany, Kmita, Alarcon, de la Pompa, & Duboule, 2001). It is possible that the somitogenesis clock is actually the *Hox* TST timer. These timers operate over similar developmental times (Jouve, Iimura, & Pourquie, 2002; Peres et al., 2006; Riedel‐Kruse et al., 2011; Wacker, Jansen, et al., 2004). However, the evidence indicates that *Hox* interactions and *BMP* also play a role in this process.

## 6 THE RELATION OF DOMAINS TO TST: WHERE AND WHEN DO THE SIGNALING PATHWAYS ACT?

Genesis of sequential A–P domains seems to depend on sequential time dependent PI, defined by interactions among *Hox* genes and putatively among more anterior determinants and between these two groups. From this viewpoint, it would seem logical that at least some domains are already set up at the temporal collinearity (TC) stage of TST. It is therefore interesting to examine the timing of the actions of signaling pathways on axial patterning. Anterior head/posterior head onset of *Wnt* action occurs in the early gastrula NO mesoderm during TC in *Xenopus* (In der Rieden et al., 2011). This early action was confirmed in primitive streak in mouse (Neijts et al., 2016). The neck/thorax transition from retinoid sensitivity to *FGF–Cdx* sensitivity was initially characterized in the late CNS (Bel Vialar et al., 2002; Godsave et al., 2008; Pownall et al., 2006). However, *Cdx* mediated sensitivity also comes on early, in mesoderm/primitive streak during TC (Neijts et al., 2017), and in fact is relevant even before gastrulation (Pillemer et al., 1998). The anterior head/posterior head switch of retinoid action is known to be important in developing neuroectoderm. However, retinoids are likely also have to a role in turning on *Hox1* during TC because retinoid loss of function downregulates not only *Hox1* but also all more posterior *Hox* genes tested. Retinoids are indeed also known to have a mesodermal function in regulating somitogenesis in presomitic/paraxial mesoderm (Vermot & Pourquie, 2005), and presumably also earlier from the beginning of somitogenesis from blastula and gastrula/ primitive streak stages in NO mesoderm (Jouve et al., 2002; Peres et al., 2006; Riedel‐Kruse, Mueller, & Oates, 2007). They presumably have an early function, affecting PI in NO mesoderm. The action of signaling pathways at decision points thus appears to reflect an extra input in addition to PI induction in the case of key decision points. Perhaps, in the *BMP* and *Hox* case of retinoids, retinoid induction of *Hox1* occurs via synergy with *BMP* and the last (most posterior) anterior head determinant in NO mesoderm (Figure 5). We cannot, however, rule out that there is somehow a role of retinoids in neuroectoderm in *Hox1* TC. Besides the familiar mesoderm to neuroectoderm signaling during neural transformation, less is known about signaling from neuroectoderm to mesoderm, which might also be important in embryogenesis (Nieuwkoop & Weijer, 1978).

## 7 HOW IS *HOX* SPECIFICITY REGULATED?

At least 13 and perhaps as many as 39 *Hox* specificities are required for PI in a diploid vertebrate, depending how many of the individual *Hox* paralogues are differently functional from each other or how many function at different times or in different places). In addition, several more specificities are required for PI if it functions in *Hox‐*independent determination of the anterior head and EAD. These specificities are determined intracellularly by homeobox proteins, and possibly other transcription factors, acting in the nucleus. The corresponding complexity of intercellular signaling during noncell autonomous *Hox–Hox* interactions could depend on the many different *Hox* genes being activated differently by specific action of different conventional signaling pathways or combinations of pathways such that a *Hox* induced ligand–receptor interaction induces a *Hox* gene via a signal transduction pathway and the induced *Hox* gene induces a second (sometimes different) ligand–receptor interaction and a second (sometimes different) signal transduction cascade (see below). This is possible, but seems to invite chaos. It is unlikely, but possible, that several different *Hox* genes could signal in the same cell without mutual interference by activating different variants of similar signaling pathways. A solution for signaling that should not be ruled out at this point is that specificity lies entirely in the specificity of intracellular *Hox–Hox* interactions in the nucleus and that noncell autonomy/signaling is regulated relatively nonspecifically via intercellular homeoprotein transport (Chatelin, Volovitch, Joliot, Perez, & Prochiantz, 1996; Joliot & Prochiantz, 2004). This type of signaling would provide an elegant and simple solution to the intercellular signaling specificity problem. It is already thought to mediate one type of monospecific *Hox–Hox* interaction during TST: noncell autonomous autoregulation during vertical signaling (Bardine et al., 2014). In PI, translocation of *Hox* proteins would have to function synergistically with the *BMP* pathway, which would need to provide no further specific A–P information except for selecting PI instead of autoregulation or PD. Synergism between conventional signaling pathways including *BMP* and homeoprotein translocation is a well‐known phenomenon (Layalle et al., 2011). It is known, in the *Drosophila* midgut mesoderm, that *BMP* induction of *Hox* genes goes along with a *BMP* positive feedback loop (Staehling‐Hampton & Hoffmann, 1994). Possibly the same happens in NO mesoderm and this is how the necessary high *BMP* level is maintained in NO mesoderm.

From the sections above, it is evident that we suspect that transcriptional control of *Hox* patterning specificity––the capacity to differentially induce or repress at least 13 different *Hox* gene classes (if these are the paralogue groups) and possibly as many as 39 (if each individual *Hox* gene is distinct)––in a diploid vertebrate is perhaps determined very little by the specificity of cell interactions because *Hox* proteins possibly travel relatively nonspecifically from cell to cell but almost entirely by the specificity of events in the nucleus. Presumably, at least in some cases, this occurs by binding of the *Hox* transcription factors to specific enhancers, resulting in either repression or activation of transcription. What are these events?

Different *Hox* proteins bind to rather similar DNA sequences. This has generated a dilemma: how is the precise developmental specificity of *Hox* gene action, which evidently exists, generated? Is it by binding of different *Hox* proteins to their own specific enhancers? There is no clear evidence that this is the case (Mann, Lelli, & Joshi, 2009). However, there is evidence that the specificity of *Hox b*inding to enhancers can be modified and enhanced by the *Hox* protein binding to members of one or both of two specific families of DNA binding homeobox containing cofactors: the *extradenticle‐Pbx* family and the *homothorax‐Meis* family. Most *Hox* proteins heterodimerise with *Pbx* proteins and it is known that binding of *Pbx‐Hox* heterodimers to their cognate response elements occurs with higher affinity and thus presumably higher specificity than binding of *Hox* monomers. The vertebrate orthologues of *Drosophila* Abd B, Hox10–13, on the other hand, less often use *Pbx* and can use Meis as a cofactor. In addition, *Homothorax‐Meis* has additional functions: it binds to *Hox‐Pbx* dimers in a tripartite complex and facilitates their entry into the nucleus as well as binding with them to enhancers (Shanmugam, Green, Rambaldi, Saragovi, & Featherstone, 1999; Shen et al., 1997). It has been proposed that *Pbx* binding converts *Hox* monomers from repressors to activators and almost all *Hox‐Pbx* complexes do indeed mediate activation responses rather than repressive ones (Mann et al., 2009).

It has been shown that response elements, including those in *Hox* genes, are often clustered in vivo (Arnone & Davidson, 1997). Recent evidence shows that multimerisation of *Hox‐Pbx* response elements does indeed regulate the effectiveness of *Hox* action in vivo (Crocker et al., 2015). In addition to cofactors for response element binding, *Hox* proteins also interact with noncooperatively binding “cooperators” (Mann et al., 2009). If co‐operating transcription factors bind to response elements sufficiently close to a *Hox* response element, they can modify its activity, either its effect (repression or activation) or the strength of the action. The direction of modification is presently not clearly predictable. Co‐operators are typically transcription factors mediating action of signaling pathways (Mann et al., 2009). Interestingly, one of the best known of these cooperating pathways is *Drosophila DPP* (Walsh & Carroll, 2007). In vertebrates, this pathway is mediated by *Smad*1, *Smad*5 and *Smad*8, the orthologues of *Drosophila Mad*1 (Figure 5 **).** A last aspect to specificity is that this would be affected by nano‐collinearity (Durston, 2018). Collinear opening of *Hox* cluster chromatin will determine which *Hox* genes are accessible for activation (or repression) due to being in a *trithorax* compartment and which are inaccessible, due to being in a *polycomb* compartment (Noordermeer et al., 2014; Soshnikova & Duboule, 2010). According to our findings above, it is likely that *Hox* cluster opening is regulated progressively by *Hox‐Hox* interactions. This fits with previous evidence for local as well as global interactions mediating chromatin opening (Durston, 2018). This could possibly account for a very specific aspect of PI: that it possibly only acts on immediate 5′‐neighboring *Hox* genes.

## 8 THE QUESTION OF GRADIENTS AND THE COMPLEXITY OF A–P PATTERNING

Patterning the body axis is obviously a complex, multistage process. *Hox* genes have different targets and are differently regulated at different developmental stages (Pavlopoulos & Akam, 2011; Weatherbee, Halder, Kim, Hudson, & Carroll, 1998). What we describe and discuss above is simply the very first stage of axial patterning. We do not know how much complexity this stage imparts. It could specify the whole axis in detail or it could simply distinguish a few different landmarks that serve as reference points for later detailed patterning, such as the transitions between domains. This brings us to patterning mechanisms. There is evidence suggesting that concentration gradients may be relevant for A–P patterning. This is possibly true for retinoids in detailed patterning of the developing hindbrain (Dupe & Lumsden, 2001; Godsave et al., 1998). There is also some evidence suggesting gradient action for FGF and *Wnt (*Kiecker & Niehrs, 2001; Lamb & Harland, 1995). These are phenomenological observations only; no mechanistic clues such as enhancers with different concentration dependencies have been identified. It is likely that retinoid gradients are specifically relevant for at least one later detailed patterning process, namely hindbrain patterning. One can, of course, not rule out that they also have other roles such as later respecifying and checking the initial pattern or acting concurrently with TST to cooperate in specifying the initial pattern. I note that all of the neck‐hindbrain *Hox* genes contain potential retinoid response elements (Mainguy et al., 2003) and that these have been confirmed to be functional early on for *Hox1* and *Hox4* genes (Gould et al., 1998; Huang et al., 2002; Langston et al., 1997; Morrisona et al., 1995; Nolte et al., 2003; Serpente et al., 2004; Zhang et al., 2000). It is not ruled out that there is a gradient aspect to the early timing mechanism described above. The Nieuwkoop activation/transformation mechanism (Nieuwkoop, 1952) showed gradient properties. Folds of neurectoderm pulled out from the early embryonic axis developed an A–P pattern with the proximal part of the fold being most posterior. It could be that whereas *Hox* genes at “decision points” first respond to a new morphogen, others in the newly founded domain also need particular concentrations of the morphogen as a cofactor, and that this interaction maintains the domain.

## 9 CONCLUSIONS

The sections above discuss the nature of early vertebrate A–P patterning and its connections with retinoids and other known A–P morphogens. We conclude from the evidence presented:

1. The familiar morphogen gradient concept is not a likely sole contender for the initial phase of A–P patterning.
2. There is a timing mechanism (TST) for initial A–P patterning. The timing mechanism depends on *BMP*/anti‐*BMP* and involves *Hox* macro‐collinearity, which is mediated by specific collinear and autonomous *Hox‐Hox* interactions.
3. The timing mechanism interacts with a higher level of control that distinguishes domains on the A–P axis.
4. The domains are distinguished by the action of different A–P signaling pathways, interacting via “decision points” at and after which they collaborate with the collinear *Hox–Hox* interactions.
5. Signaling pathways can also act in other ways, for example as collaborators that co‐mediate noncell autonomous *Hox–Hox* interactions.
6. The existence of a second early TST mechanism coupled to early A–P patterning (somitogenesis) poses an interesting problem yet to be resolved. Somitogenesis interacts with *Hox* TST.
7. The question of how *Hox* specificity is regulated poses another interesting problem for future investigation.
8. The A–P patterning process is complex and time dependent. I deal here only with the initial phase. There are other important phases, which presumably have their own specific mechanisms.
9. This review provides some novel clues as to how the vertebrate A–P pattern is set up. There is still a lot to be done.
